# Proteomic Profiling of γ-Secretase Substrates and Mapping of Substrate Requirements

**DOI:** 10.1371/journal.pbio.0060257

**Published:** 2008-10-21

**Authors:** Matthew L Hemming, Joshua E Elias, Steven P Gygi, Dennis J Selkoe

**Affiliations:** 1 Center for Neurologic Diseases, Brigham and Women's Hospital and Harvard Medical School, Boston, Massachusetts, United States of America; 2 Department of Cell Biology, Harvard Medical School, Boston, Massachusetts, United States of America; University of California San Francisco/Howard Hughes Medical Institute, United States of America

## Abstract

The presenilin/γ-secretase complex, an unusual intramembrane aspartyl protease, plays an essential role in cellular signaling and membrane protein turnover. Its ability to liberate numerous intracellular signaling proteins from the membrane and also mediate the secretion of amyloid-β protein (Aβ) has made modulation of γ-secretase activity a therapeutic goal for cancer and Alzheimer disease. Although the proteolysis of the prototypical substrates Notch and β-amyloid precursor protein (APP) has been intensely studied, the full spectrum of substrates and the determinants that make a transmembrane protein a substrate remain unclear. Using an unbiased approach to substrate identification, we surveyed the proteome of a human cell line for targets of γ-secretase and found a relatively small population of new substrates, all of which are type I transmembrane proteins but have diverse biological roles. By comparing these substrates to type I proteins not regulated by γ-secretase, we determined that besides a short ectodomain, γ-secretase requires permissive transmembrane and cytoplasmic domains to bind and cleave its substrates. In addition, we provide evidence for at least two mechanisms that can target a substrate for γ cleavage: one in which a substrate with a short ectodomain is directly cleaved independent of sheddase association, and a second where a substrate requires ectodomain shedding to instruct subsequent γ-secretase processing. These findings expand our understanding of the mechanisms of substrate selection as well as the diverse cellular processes to which γ-secretase contributes.

## Author Summary

All cells face the challenge of removing transmembrane proteins from the lipid bilayer for the purpose of signaling or degradation. One molecular solution to this problem is the multiprotein enzyme complex γ-secretase, which is able to hydrolyze several known transmembrane proteins within the hydrophobic lipid environment. Due to its central role in the pathogenesis of Alzheimer disease, modulation of γ-secretase activity has become a therapeutic goal. However, the number and diversity of proteins that can be cleaved by this protease remain unknown, and the attributes that target these proteins to γ-secretase are unclear. In this study, we used an unbiased approach to substrate identification and surveyed the proteome for targets of γ-secretase. Of the thousands of proteins detectable, only a relative few were substrates of γ-secretase, all of which were type I transmembrane proteins. In addition to validating several of these novel substrates, we compared them to other proteins that we identified as nonsubstrates and determined that there are specific domains that can activate or inhibit γ-secretase processing. These findings should advance our understanding of the many cellular processes regulated by γ-secretase and may offer insights into how γ-secretase can be exploited for therapeutic purposes.

## Abstract 3

Using an unbiased quantitative proteomics approach, novel substrate targets for the protease γ-secretase are identified and analyzed to determine which domains enable their cleavage.

## Introduction

In the recently discovered process of regulated intramembrane proteolysis, activated transmembrane proteins are liberated from the lipid bilayer in a two-step mechanism. The first cleavage by a class of proteases dubbed secretases or sheddases releases the ectodomain, leaving the protein with a short lumenal stub, a transmembrane domain, and a cytoplasmic domain. The second scission occurs when a protease uses an unusual active site within the hydrophobic lipid environment to recognize and cleave the truncated target protein, releasing both the lumenal fragment and the cytoplasmic domain from the membrane. The released intracellular domain (ICD) may then signal as a transcription factor or by other means [1,2]. This process was first elucidated in studies of the pathogenesis of Alzheimer disease, in which the amyloid precursor protein (APP) is initially cleaved by β-secretase to generate an APP C-terminal fragment (CTF) that is subsequently cleaved by the intramembrane aspartyl protease γ-secretase, releasing amyloid β-protein (Aβ) from the membrane. Secreted Aβ initiates the amyloidogenic cascade that is widely believed to drive pathogenesis [3].

γ-secretase is a multiprotein complex consisting of presenilin (PS), nicastrin, Aph-1, and Pen-2, with PS containing the two catalytic aspartates that mediate peptide bond scission [4]. PS is synthesized as a holoprotein that is post-translationally cleaved into an N-terminal fragment (NTF) and a CTF, which remain bound as a heterodimer. More than 160 different missense mutations have been identified within the two human presenilin genes that cause an aggressive, early-onset form of Alzheimer disease, largely by producing longer and thus more aggregation prone species of Aβ [5]. For its key role in Aβ generation, γ-secretase has become a principal target for Alzheimer disease therapeutics aimed at inhibiting or modulating the protease's activity. In addition, γ-secretase inhibition may prove therapeutic for some forms of cancer by decreasing intracellular signaling molecules, e.g. the Notch ICD, that are generated by γ cleavage [6].

Through extensive investigation, principally of the prototypical substrates Notch and APP, a general model of γ-secretase function and activity has emerged [4]. γ-secretase processing is preceded by shedding of the substrate's ectodomain by either an α- or β-secretase, generating a CTF with a short N-terminal extracellular domain. Once this conversion to a CTF has occurred, the γ-secretase complex can bind the substrate and then translocate it to the active site, where intramembrane proteolysis can occur within an interior hydrophilic chamber [7,8]. There remain several major gaps in our understanding of γ-secretase proteolysis. It is unclear what number and spectrum of substrates are processed by γ-secretase. From the existing candidate-based substrate studies, it has been proposed that γ-secretase may function as a type of “proteasome of the membrane,” with loose substrate specificity [9]. It is thought that γ-secretase can only cleave type I transmembrane proteins. However, substrates of other topologies have been proposed [10,11], and preference for type I proteins has never been addressed in an unbiased manner. Finally, it is unclear which regions of a substrate are important for binding and subsequent proteolysis by γ-secretase, and whether a sheddase may, beyond reducing ectodomain size, contribute to substrate specificity.

To address these issues, we have performed a novel unbiased proteomic screen to identify the range of substrates in the proteome that are regulated by γ-secretase processing. Coupling a selective γ-secretase inhibitor with the method of stable isotope labeling with amino acids in cell culture (SILAC) and subsequent mass spectrometry, we have identified a relatively small cohort of novel γ-secretase substrates among thousands of other proteins that are unchanged by γ-secretase inhibition. In agreement with the general model of γ-secretase processing, all substrates we identified were type I transmembrane proteins. Using genetic and pharmacological manipulations, we validated a subset of substrates and nonsubstrates and confirmed that γ-secretase cleavage is preceded by ectodomain shedding. By generating chimeric proteins containing substrate and nonsubstrate regions, we identified two potentially independent mechanisms for targeting a protein for proteolysis. Finally, we determined that γ-secretase binding of a truncated type I protein is not sufficient to induce proteolysis, and that permissive transmembrane and cytoplasmic domains are required for γ-secretase cleavage to occur. These results demonstrate that γ-secretase regulates a relatively small subset of the membrane proteome, and that substrates have specific determinants that enable their recognition and proteolysis.

## Results

### Identification of γ-Secretase Substrates Using Quantitative Proteomics and γ-Secretase Inhibition

The development of tools to compare proteomes quantitatively has enabled identification of phosphorylation cascades [12], DNA damage responses [13], and changes in tumor cell lines [14]. The SILAC method takes advantage of the ability of mass spectrometry to differentiate between heavy and light isotopic variants. By metabolically labeling cells with amino acids containing either heavy or light isotopes, the entire proteome can be quantitatively compared, and differences arising from genetic disparity or experimental treatments can be determined. In the present study, we sought to identify substrates of γ-secretase using SILAC coupled with treatment by a selective γ-secretase inhibitor.

**Figure 1:**
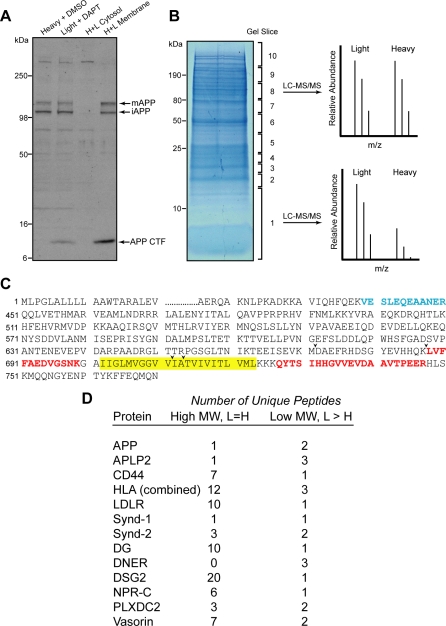
Identification of γ-Secretase Substrates HeLa cells were labeled using growth media containing heavy or light forms of arginine and lysine. (A) Cells grown with the light label were subjected to the γ-secretase inhibitor DAPT, which leads to accumulation of the APP CTF compared to the heavy-labeled control cells (lanes 1 and 2). Equal numbers of cells from the Light+DAPT and Heavy+DMSO conditions were combined and fractionated into cytosolic and membrane proteins (lanes 3 and 4). (B) One hundred micrograms of the combined Light+DAPT and Heavy+DMSO membrane fraction were separated by SDS-PAGE. The gel was divided into ten horizontal slices and each subjected to trypsinization and LC-MS/MS. In quantitative comparisons, substrates would be expected to have equal relative abundance of full-length proteins (upper spectrum) of the Light+DAPT and Heavy+DMSO conditions; whereas truncated fragments normally cleaved by γ-secretase (CTFs) would show an increased abundance of light-labeled peptides in the presence of DAPT (lower spectrum). (C) Portion of the APP primary sequence, with a tryptic peptide identified from the higher–molecular weight (MW) portion of the gel and unchanged in relative abundance indicated in blue. Red indicates tryptic peptides found in the lower-MW portion of the gel that were more abundant in the Light+DAPT condition. The transmembrane sequence is highlighted in yellow, and arrowheads indicate the β-, α-, γ-40, and γ-42 secretase cleavage sites (from left to right). APP-770 amino acid numbering is indicated on the left. (D) List of all substrates identified through the proteomics screen, with the number of higher-MW, equal-abundance peptides in the left column and the number of lower MW peptides that accumulate from DAPT treatment in the right column. Note that all unique HLA peptides identified were combined in the table due to high sequence redundancy among the different HLA isoforms.

HeLa cells were labeled with light or heavy lysine and arginine. Once labeled, the γ-secretase inhibitor DAPT was applied to the light labeled cells (Light+DAPT) and control DMSO solvent to the heavy labeled cells (Heavy+DMSO). After this 16 h treatment, equal numbers of cells were combined and then fractionated. As evidence of successful γ-secretase inhibition, we examined endogenous APP levels by Western blot (Figure 1A). In whole-cell lysates (lanes 1 and 2) DAPT treatment did not change the levels of full-length APP, but as expected increased the levels of the APP CTF, the immediate substrate of γ-secretase. Proteins from the combined Light+DAPT and Heavy+DMSO treatment conditions were fractionated into cytosolic and membrane fractions, with the APP and APP CTFs found in the membrane fraction and absent from the cytosol. Purified membrane proteins were separated by SDS-PAGE and stained with Coomassie (Figure 1B). For mass spectrometry analysis, the lane was divided into ten horizontal slices, and each subjected to tryptic digestion and LC-MS/MS (see Materials and Methods for details).

The expected spectral pattern for full-length APP and APP CTF peptides, as well as other putative γ-secretase substrates, is shown in schematic form in Figure 1B. Peptides derived from full-length proteins would not be expected to change in relative abundance in response to γ-secretase inhibition (upper spectrum), regardless of whether they are true substrates or nonsubstrates. In contrast, peptides derived from γ-secretase substrates, following ectodomain shedding that reduces the protein's molecular weight, should be higher in relative abundance in the light-labeled condition treated with DAPT (lower spectrum). Data arising from all quantitative peptide comparisons were analyzed for this spectral pattern to identify individual γ-secretase substrates expressed under endogenous conditions. In total, over 16,400 peptides representing more than 2,500 proteins were quantitatively compared (Table S1).

The APP protein sequence is shown in Figure 1C to exemplify the findings of this proteomic analysis. An APP tryptic peptide derived from the higher–molecular weight region of the gel and present in equal abundance under the two labeling conditions is indicated in blue, and two peptides from the lower–molecular weight region of the gel with significantly increased abundance in the Light+DAPT condition are indicated in red. As expected for APP, the peptides enriched in the Light+DAPT condition map to the C-terminal region of the protein, which remains embedded in the membrane after ectodomain shedding and represents the substrate for γ-secretase. Figure 1D displays all the proteins identified by this quantitative proteomic screen that fit the criteria for a potential γ-secretase substrate. Listed next to the protein in the left column are the higher–molecular weight, equal-abundance peptides, and in the right column are the relatively lower–molecular weight peptides enriched by DAPT treatment. APP and its homolog amyloid precursor-like protein 2 (APLP2) [15], as well as CD44 [16], are previously described γ-secretase substrates. The other APP homolog, APLP1, was not identified in this screen, consistent with it being expressed in the nervous system [17] and thus not present in the epithelial HeLa cell line. Several novel substrates were identified in this screen that have closely related homologues previously identified as γ-secretase substrates: the human leukocyte antigen (HLA) [18], the low-density lipoprotein receptor (LDLR) [19], and syndecan-1 and −2 (Synd) [20]. The remaining novel γ-secretase substrates we identified are: dystroglycan (DG), the Delta/Notch-like EGF-related receptor (DNER), desmoglein-2 (DSG2), natriuretic peptide receptor-C (NPR-C), plexin domain-containing protein 2 (PLXDC2), and vasorin. Table S2 lists the sequence and quantitative information of the peptides derived from these putative γ-secretase substrates.

### Validation of Identified γ-Secretase Substrates and Nonsubstrates Using Genetic and Pharmacological Systems

**Figure 2:**
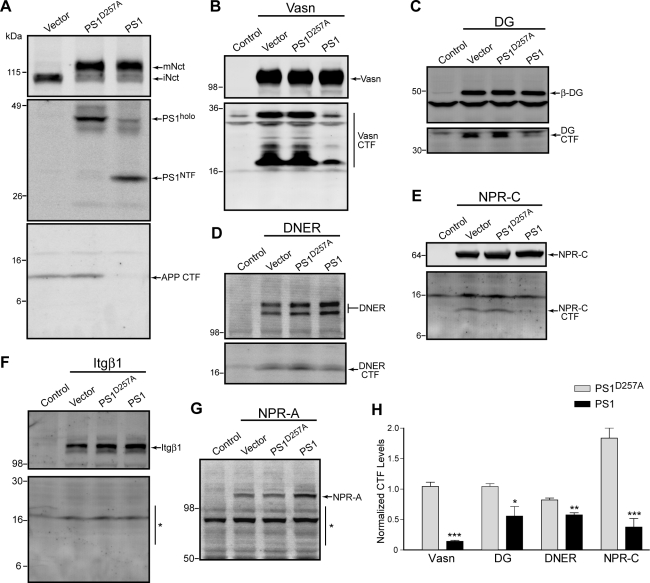
Validation of γ-Secretase Substrates Using a Genetic System (A) Fibroblasts derived from *Presenilin1/2* null mice were transduced to stably express a control empty vector, a catalytically inactive PS1-D257A construct, or the wild-type PS1 construct. PS1-D257A and wild-type PS1 enabled the formation of the γ-secretase complex, as indicated by the maturation of nicastrin (mNct) from incompletely glycosylated immature nicastrin (iNct, top panel). The PS1-NTF and a small amount of PS1-holoprotein were observed with wild-type PS1 expression, as expected, but cells expressing PS1-D257A were unable to convert PS1-holo into PS1-NTF (middle panel). Endogenous APP CTFs (bottom panel) indicate that proteolytically active γ-secretase was present only in the wild-type PS1 condition. (B–E) In empty vector, PS1-D257A, and wild-type PS1 cells, FLAG-tagged candidate substrates were stably expressed to validate their processing by γ-secretase: (B) vasorin (Vasn), (C) dystroglycan (DG), (D) Delta/Notch-like EGF-related receptor (DNER), and (E) natriuretic peptide receptor-C (NPR-C). Two type I transmembrane proteins found in the screen not to be processed by γ-secretase—(F) integrin β-1 (Itgβ1) and (G) natriuretic peptide receptor-A (NPR-A)—were similarly analyzed and validated, with the predicted size of a theoretical CTF indicated with a vertical line and asterisk. (H) Quantitative changes in substrate CTF levels normalized to the empty vector control. For comparisons between PS1-D257A and PS1 conditions, *p < 0.05, **p < 0.01, ***p < 0.001.

To confirm that the proteins identified in the SILAC proteomic screen are indeed γ-secretase substrates, DG, DNER, DSG2, NPR-C, PLXDC2, and vasorin were each cloned and C-terminally tagged with a FLAG epitope. To also validate two of the many proteins identified by the screen that were not implicated as γ-secretase substrates, the proteins integrin β-1 (Itgβ1) and natriuretic peptide receptor-A (NPR-A) were similarly cloned and FLAG-tagged. These two apparent nonsubstrates were chosen based on their similarities to known substrates, including type I transmembrane topology and function in cell adhesion (Itgβ1) or as a peptide receptor (NPR-A). As a genetic method of validation (Figure 2), we used a fibroblast cell line that contains deletions of the *Presenilin-1* and *-2* genes and thus completely lacks γ-secretase activity. In this PS double-knockout (PS-DKO) cell line, we expressed one of three constructs by viral transduction to generate a substrate validation system: (1) a control cell line expressing just empty vector; (2) a cell line expressing a catalytically inactive PS construct with one active site aspartate mutated to alanine (PS1-D257A), which thus restores γ-secretase complex formation but prevents catalytic activity; and (3) a cell line expressing wild-type PS1 that forms a proteolytically active γ-secretase complex (Figure 2A). Due to low expression of some proteins, not all cloned substrates could be tested in these PS-DKO cells, but they were validated separately by DAPT inhibitor treatment (see below).

Expression of either PS1-D257A or wild-type PS1 allowed the formation of the γ-secretase complex, as confirmed by the mature glycosylation of nicastrin [21] (Figure 2A, top panel). Probing for PS1 by Western blot with an N-terminally directed antibody (middle panel) showed both PS1 holoprotein and NTF in the wild-type PS1 cells, whereas the PS1-D257A construct remained as a holoprotein, failing to be post-translationally cleaved into NTF and CTF. These data confirm that PS holoprotein processing into NTF and CTF occurs by an endoproteolytic mechanism dependent on PS activity. As a readout for establishment of γ-secretase activity, these various cell lines were probed for the presence of endogenous APP CTFs (lower panel), whose levels were reduced in the PS1-expressing cells, thus indicating functional γ-secretase activity only with expression of wild-type presenilin.

**Figure 3:**
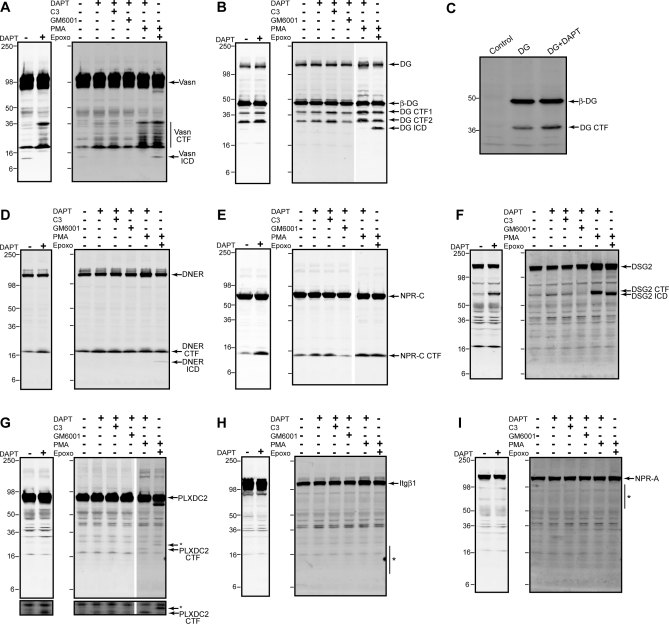
Pharmacological Characterization of γ-Secretase Substrate Processing HEK cells stably expressing the indicated constructs were treated for 16 h with DAPT (+) or DMSO vehicle alone (–) (left panels), or for 6–16 h with DAPT alone or in combination with the β-secretase inhibitor C3, the metalloprotease inhibitor GM6001, the phorbol ester PMA, and the proteasome inhibitor epoxomicin (right panels). The constructs expressed are (A) vasorin, (B) DG, (D) DNER, (E) NPR-C, (F) DSG2, (G) PLXDC2, (H) Itgβ1, and (I) NPR-A. (C) DG was also expressed in HeLa cells, revealing just a single CTF that again accumulated with DAPT treatment. The asterisk in (G) indicates a PLXDC2 fragment that accumulated with simultaneous epoxomicin and DAPT treatment (data not shown). A quantitative analysis of these experiments is shown in Figure S1.

As a second means of validation, the cloned substrates and nonsubstrates were expressed stably in HEK cells, and the presence or absence of regulation by γ-secretase was confirmed by treatment with DAPT (Figure 3). In further analyses of these cells, we searched for evidence of the sheddase responsible for ectodomain secretion by applying the β-secretase inhibitor C3, the metalloprotease (α-secretase) inhibitor GM6001, or the phorbol ester PMA, which stimulates ectodomain shedding. To probe for the γ-secretase–mediated release of intracellular domains, many of which have been shown to be degraded rapidly by the proteasome [18,22–24], we treated cells with the proteasome inhibitor epoxomicin. The genetic (Figure 2) and pharmacological (Figure 3) experiments will be described together for each potential substrate or nonsubstrate.

Vasorin is an inhibitor of TGF-β signaling and may play a role in vascular remodeling [25]. In the PS-DKO cells, vasorin CTF levels were potently reduced by active γ-secretase (Figure 2B and 2H). Vasorin achieved high overexpression in HEK cells, with significant CTF accumulation in response to DAPT treatment, and a reduction in CTF levels with a 6-h GM6001 treatment, indicating the vasorin sheddase to be a metalloprotease (Figures 3A and S1A). A prominent vasorin ICD was observed with vasorin expression, which was further stabilized by proteasome inhibition (Figure 3A, far right lane). In both the PS-DKO and HEK cells, several distinct vasorin CTF bands were observed. Similarly, when expressed at comparatively low levels in HeLa cells, multiple vasorin CTF bands were still generated (unpublished data). This CTF pattern is unlikely to be due to multiple sheddase cleavages (see below), but may be due to post-translational modification or SDS-stable multimer formation. Of interest, we noticed that stable vasorin expression in HEK cells altered cell morphology and reduced cell adherence (Figure S1I), although it did not produce overt cytotoxicity. Such a morphological change was not observed upon expression of any of the other γ-secretase substrates.

DG, a member of the multiprotein dystrophin–glycoprotein complex, provides a physical connection between the extracellular matrix and the intracellular cytoskeleton [26] and is implicated in several diseases, including muscular dystrophies [27]. DG is synthesized as a long precursor protein and is post-translationally cleaved into an extracellular α-DG fragment that noncovalently associates with the membrane-anchored β-DG fragment [28]. In our PS-DKO cell lines, β-DG was observed to undergo shedding to generate a DG CTF, whose levels were then regulated by γ-secretase activity (Figure 2C and 2H). In the HEK cells, DAPT treatment led to a significant increase in two distinct DG CTFs, one of which was produced by metalloprotease cleavage (Figures 3B and S1B–S1D). A DG ICD was observed without DAPT treatment, which significantly accumulated with proteasome inhibition (Figure 3B, right panel). To compare the complex banding pattern observed in HEK cells, suggesting more than one ectodomain proteolytic pathway, we also analyzed DG processing in HeLa cells. When DG was expressed in HeLa cells, only a single CTF was produced from β-DG, and it accumulated upon γ-secretase inhibition (Figures 3C and S1B). Thus, different cell types apparently use different β-DG secretory processing pathways.

DNER is a Notch ligand expressed in neurons and involved in cerebellar development and function [29,30]. In the PS-DKO cell lines, full-length DNER was expressed as a doublet, perhaps arising from differential glycosylation, and produced a CTF that was regulated by γ-secretase (Figure 2D and 2H). In HEK cells, DNER expression resulted in the production of a CTF whose levels also increased with DAPT treatment. After a 16-h treatment, the metalloprotease inhibitor GM6001 modestly reduced CTF levels by inhibiting α-secretase mediated ectodomain shedding (Figure S1E). The DNER ICD could be detected upon inhibition of the proteasome (Figure 3D, far right lane).

NPRs bind to circulating natriuretic peptides (atrial natriuretic peptide, brain natriuretic peptide, and C-type natriuretic peptide) [31]. NPR-A and NPR-B contain a guanylate cyclase domain, and upon binding natriuretic peptides generate cyclic GMP. NPR-C, while similar to NPR-A and -B in the function of its extracellular domain, has a shortened cytoplasmic domain with no guanylate cyclase activity. Thus, with no identified signaling function [32], NPR-C has been termed a “clearance receptor” whose principal role may be to remove excess natriuretic peptides [33,34]. However, recent studies have identified regions in the cytoplasmic tail of NPR-C that modulate G-protein activity [35], suggesting that NPR-C's short cytoplasmic tail has a signaling function. In our PS-DKO cell lines, expression of full-length NPR-C resulted in an NPR-C CTF, the levels of which were significantly regulated by γ-secretase activity (Figure 2E and 2H). Expression in HEK cells revealed robust CTF accumulation with DAPT treatment, and metalloprotease inhibition by 16-h GM6001 treatment reduced CTF levels (Figures 3E and S1F).

Desmogleins are structural components of desmosomes, which form intercellular junctions and direct tissue morphogenesis [36]. Overexpression of DSG2 has been noted in several forms of cancer, and transgenic overexpression of DSG2 drives tumorigenesis by altering multiple signaling pathways [37]. In HEK cells, DSG2 expression produced a full-length protein and a CTF that was processed by γ-secretase. CTF levels decreased significantly upon 6-h treatment with either C3 or GM6001, indicating that both β-secretase and an α-secretase–like metalloprotease can shed the DSG2 ectodomain (Figures 3F and S1G). The DSG2 ICD was observed when its degradation was prevented by the proteasome inhibitor epoxomicin (Figure 3F, far right lane).

PLXDC2 is a poorly characterized protein expressed in the developing nervous system [38] and found to be up-regulated in cancerous tissue [39]. Full-length PLXDC2 is robustly expressed in HEK cells, though the levels of CTF produced by ectodomain shedding are relatively low. Shedding is apparently due to a metalloprotease, as a 16-h GM6001 treatment significantly reduces CTF levels, while the CTF levels increase with PMA treatment (Figures 3G and S1H).

In addition to the novel substrates discussed above, analysis of proteins that we identified to not be processed by γ-secretase may yield insight into substrate requirements. Integrins are cell adhesion molecules that also play substantial roles in signal transduction. All integrin isoforms are single-pass membrane proteins that form αβ heterodimers [40]. Itgβ1 achieved modest expression in PS-DKO cells (Figure 2F), and stronger expression in HEK cells (Figure 3H), although no CTF was observed and protein levels were not modulated by sheddase activators or inhibitors (Figure 3H, right panel). We also analyzed endogenous Itgβ1 levels in the PS-DKO cells using a C-terminally directed antibody, and again found no evidence for CTF production (unpublished data). The second nonsubstrate examined was NPR-A, and despite being in the same family as NPR-C, NPR-A did not yield a CTF and was not proteolytically regulated by sheddase or γ-secretase activity, both in PS-DKO cells (Figure 2G) and HEK cells (Figure 3I). Thus, as first identified in our unbiased proteomics screen, these two proteins are not regulated by γ-secretase processing, despite having type I topology and functions in common with some other known γ-secretase substrates.

### Chimeric Constructs of Integrin and Vasorin Identify a Role for the Transmembrane and Cytoplasmic Domains in γ-Secretase Proteolysis

**Figure 4:**
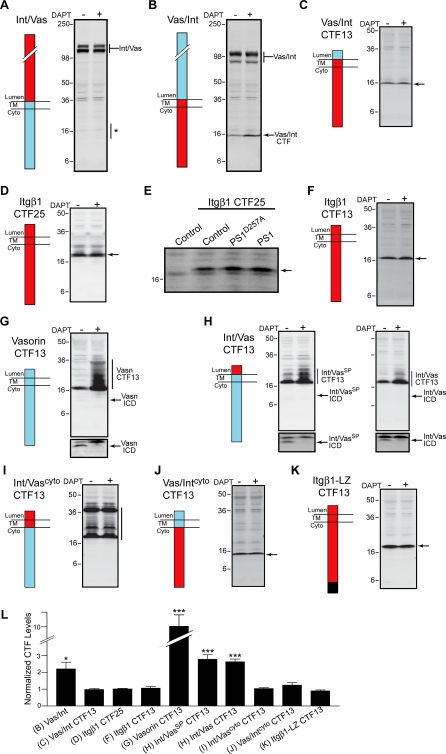
Processing of Chimeric Constructs of Integrin and Vasorin The lumenal, transmembrane, and/or cytoplasmic domains of integrin (a nonsubstrate; red) and vasorin (a γ-secretase substrate; blue) were interchanged to determine how proteins are targeted for γ-secretase processing. HEK cells expressing the constructs were treated with DAPT or vehicle alone for 16 h. In the case of Itgβ1 CTF25, (E) PS-DKO fibroblast cells were also analyzed as in Figure 2. (A and B) The full-length ectodomains of integrin (Int) and vasorin (Vas) were exchanged, yielding a CTF regulated by γ-secretase only from the Vas/Int chimera. (C–K) Recombinant CTFs were generated by fusing the protein's signal peptide to the lumenal domain either 25 (CTF25) or 13 (CTF13) residues N-terminal to the transmembrane domain. Integrin CTF constructs with an ectodomain of 25 residues (D and E) or 13 residues (F) were not processed by γ-secretase. This was also true of the Itgβ1-LZ CTF13 construct that has a leucine zipper at its C-terminus to facilitate homo-dimerization (K). (C, I, and J) Chimeric CTFs containing an integrin transmembrane and/or cytoplasmic domain were not processed by γ-secretase. (G and H) Vasorin CTF13 and chimeric constructs containing both the vasorin transmembrane and cytoplasmic domains (Int/Vas CTF13) are processed by γ-secretase to produce ICDs (lower panel, darker exposure), and CTFs accumulate in the presence of DAPT. Note that in (H), Int/Vas CTF13 chimeras were generated using either the vasorin signal peptide (Int/Vas^SP^ CTF13, left panel) or the integrin signal peptide (Int/Vas CTF13, right panel), producing similar results. (L) The fold accumulation of CTFs in response to DAPT versus DMSO control; *p < 0.05, ***p < 0.001.

Our finding that Itgβ1 and NPR-A are not γ-secretase substrates suggests that protease activity is directed only at specific type-I transmembrane proteins. In an effort to establish which regions within a substrate are important for γ-secretase recognition and proteolysis, we used recombinant methods to fuse the domains of a γ-secretase substrate (vasorin) and a nonsubstrate (Itgβ1), thereby producing various chimeric type I proteins. By expressing these chimeras in HEK cells and inhibiting γ-secretase activity with DAPT, it becomes feasible to determine which regions of a protein are permissive or inhibitory to γ-secretase processing. First, we examined chimeras of full-length proteins. A construct containing the full-length ectodomain of integrin fused to the transmembrane and cytoplasmic domains of vasorin (Int/Vas) was expressed at high levels, although no CTF was produced. This is presumably due to the observed lack of ectodomain shedding of the integrin extracellular domain (above), and thus Int/Vas is not a substrate of γ-secretase (Figure 4A), even though the transmembrane domain of intact vasorin undergoes robust γ cleavage. In contrast, when the ectodomain of vasorin is fused to the transmembrane and cytoplasmic domain of Itgβ1 (Vas/Int), ectodomain shedding occurs to produce a CTF that is processed by γ-secretase (Figure 4B). Thus, ectodomain shedding is a requirement of γ-secretase processing, even when a protein contains a transmembrane and cytoplasmic domain normally recognized and cleaved by γ-secretase.

To bypass the requirement for ectodomain shedding, chimeric CTFs can be produced directly by fusing the signal peptide to the ectodomain 13 amino acids before the transmembrane domain (designated CTF13). Signal peptide removal produces a protein that resembles identically a CTF generated by sheddase cleavage [41]. Interestingly, the Vas/Int CTF13 protein was not regulated by γ-secretase (Figure 4C). This is in contrast to the Vas/Int CTF derived from ectodomain shedding of the full-length Vas/Int (Figure 4B). Taken together with data from other chimeric CTFs discussed below, these data suggest that there may be an alternative pathway for γ-secretase recognition of a CTF, in addition to the canonical stepwise cleavages by a sheddase and then γ-secretase, which may depend on the context in which the CTF is generated and presented to γ-secretase (see Discussion).

It has been hypothesized that due to the lack of a consensus recognition sequence in the known γ-secretase substrates, the protease has loose sequence specificity and simply requires that a protein's ectodomain is shed to enable γ-secretase processing. To test this hypothesis, we made artificial CTFs of Itgβ1, which normally does not have its ectodomain shed and is not regulated by γ-secretase cleavage (Figures 2F and 3H). We first examined Itgβ1 CTF25, which after signal peptide removal should produce a CTF with an ectodomain containing the first 25 amino acids of the integrin lumenal domain. Expression in HEK cells with versus without DAPT treatment (Figure 4D) and in the PS-DKO cell system (Figure 4E) produced no evidence for processing of Itgβ1 CTF25 by γ-secretase. Similar results were observed for the Itgβ1 CTF13, which has a shorter ectodomain as would be generated by an α-secretase (Figure 4F). Thus, γ-secretase requires that a substrate has features more than just a short ectodomain. In this regard, dimerization of CTFs has been hypothesized to be important for γ-secretase processing of APP [42] and activated tyrosine kinase receptors [43], although forcing dimerization of the ectodomain can inhibit γ cleavage [44]. To investigate whether intracellular dimerization of a CTF is sufficient to confer γ-secretase cleavage, we appended the dimerization-inducing leucine zipper domain to the C terminus of Itgβ1 CTF13 to produce Itgβ1-LZ CTF13. However, Itgβ1-LZ CTF13 levels were not changed by DAPT treatment (Figure 4K), suggesting that homo-dimerization is not sufficient to promote γ-secretase proteolysis.

The vasorin CTF13, as expected from previous results with the full-length vasorin protein (Figures 2B and 3A), was strongly regulated by γ-secretase processing in that DAPT treatment increased vasorin CTF13 levels approximately 10-fold (Figure 4G and 4L). Also consistent with γ-secretase cleavage of the vasorin CTF13, a vasorin ICD was observed in the absence of DAPT (Figure 4G, lower panel). Of note, accumulated vasorin CTF13 produced higher–molecular weight bands similar to those seen with expression of the full-length protein, again suggesting either complex post-translational modification or a very stable multimerization of the CTF. A CTF chimera of integrin and vasorin composed of a short integrin ectodomain plus the vasorin transmembrane and cytoplasmic domains (Int/Vas CTF13) was processed by γ-secretase (Figure 4H), with significant increases in CTF levels caused by DAPT treatment (Figure 4L). Int/Vas CTF13 constructs also produced a detectable ICD following γ-secretase cleavage (Figure 4H, lower panel), which further accumulated with proteasome inhibition (unpublished data). That Int/Vas CTF13 (Figure 4H), but not the full-length Int/Vas (Figure 4A), was processed by γ-secretase further supports ectodomain shedding as a necessary event occurring prior to a substrate's recognition by γ-secretase.

Integrin and vasorin CTF chimeras that contain only the vasorin cytoplasmic domain (Int/Vas^cyto^ CTF13) (Figure 4I) or only the vasorin lumenal and transmembrane domains (Vas/Int^cyto^ CTF13) (Figure 4J) were not substrates of γ-secretase. Due to the higher–molecular weight bands observed in the Int/Vas^cyto^ CTF13 (Figure 4I), the vasorin cytoplasmic domain is likely the site of post-translational modification that produces the banding pattern also observed in natively produced vasorin CTF, vasorin CTF13, and Int/Vas CTF13. Taken together, the data suggest that both a permissive transmembrane and a permissive cytoplasmic domain are necessary to confer γ-secretase cleavage upon a substrate, and that the ectodomain must be short but does not by itself dictate substrate processing.

### Chimeric Constructs of NPR-A and NPR-C Demonstrate an Inhibitory Role of the NPR-A Cytoplasmic Domain in γ-Secretase Processing

**Figure 5:**
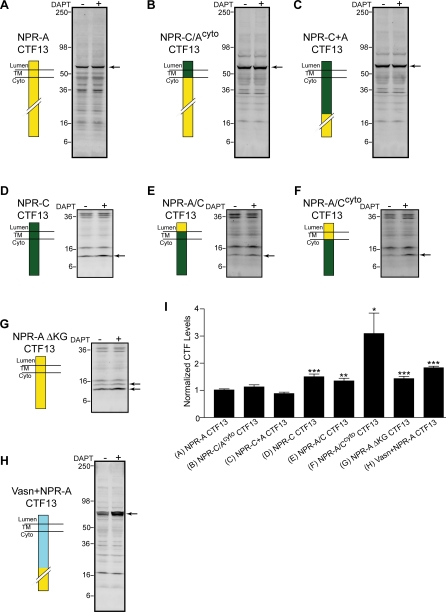
Chimeric Constructs of NPR-A and NPR-C Regions of NPR-C (green) and NPR-A (yellow) were recombined to generate chimeric CTFs. (A–C) The NPR-A CTF13 (A) and other NPR constructs containing the full-length NPR-A cytoplasmic domain, whether fused to the NPR-C lumenal and transmembrane domains (NPR-C/A^cyto^ CTF13) (B) or fused to the end of NPR-C (NPR-C+A CTF13) (C), were not regulated by γ-secretase. (D–F) The levels of NPR-C CTF13 (D) and NPR-A/C chimeras containing the lumenal domain of NPR-A (NPR-A/C CTF13) (E) or the lumenal and transmembrane domains of NPR-A (NPR-A/C^cyto^ CTF13) (F) were significantly elevated in response to DAPT, indicating their processing by γ-secretase. (G) NPR-A ΔKG CTF13, which lacks the characterized NPR-A cytoplasmic domains, was also elevated by γ-secretase inhibition. By Western blot, this construct appears as two distinct bands. (H) Vasn+NPR-A CTF13 levels were increased modestly with DAPT treatment, but relative accumulation of the CTF was far less than vasorin CTF13 (Figure 4G). (I) The fold accumulation of CTFs arising from DAPT treatment versus DMSO control; *p < 0.05, **p < 0.01, ***p < 0.001.

To determine why NPR-C is regulated by γ-secretase processing but not by the NPR-A protein, despite their being closely related receptors, we generated various chimeric constructs in a similar manner as those described above. The NPR-A CTF13 construct itself, like Itgβ1 CTFs, was not processed by γ-secretase (Figure 5A); again demonstrating that more than ectodomain shedding is required for a protein to be a γ-secretase substrate. Moreover, when the large intracellular domain of NPR-A (containing the kinase homology, hinge, and guanylate cyclase domains [45]) was attached to either the NPR-C lumenal and transmembrane domains (NPR-C/A^cyto^ CTF13) (Figure 5B), or fused to the C terminus of the NPR-C CTF (NPR-C+A CTF13) (Figure 5C), the resulting constructs were not processed by γ-secretase (quantified in Figure 5I).

In contrast to the chimeras bearing the NPR-A C terminus, NPR-C CTF13 levels were increased with DAPT treatment (Figure 5D), as expected for this previously demonstrated substrate (Figure 2E and 2H). Addition of the lumenal NPR-A domain (NPR-A/C CTF13) (Figure 5E) or both the NPR-A lumenal and transmembrane domains (NPR-A/C^cyto^ CTF13) (Figure 5F) did not perturb the ability of the construct to be regulated by γ-secretase (quantified in Figure 5I). That these three CTFs bearing the NPR-C cytoplasmic domain are processed, but not the constructs bearing the NPR-A cytoplasmic domain, suggested an inhibitory role of the NPR-A cytoplasmic tail in γ-secretase processing. To test this hypothesis, we truncated the NPR-A C terminus by removing the three domains (kinase, hinge, and guanylate cyclase) to produce NPR-A ΔKG CTF13, which has a cytoplasmic tail of similar size to that of NPR-C. Treatment of cells expressing this construct with DAPT showed that NPR-A ΔKG CTF13 was regulated by γ-secretase (Figure 5G and 5I) to a similar extent as NPR-C CTF13. These data demonstrate that, while the NPR-A transmembrane and juxtamembrane domains are permissive as substrates, the C-terminal cytoplasmic tail of NPR-A is not permissive of γ-secretase processing. This inhibition is not simply due to the large size of the NPR-A cytoplasmic domain, however, because the γ-secretase substrate desmoglein-2 contains a cytoplasmic domain of similarly large size (Figure 3F).

To determine if the processing of a substrate more potently regulated by γ-secretase could be inhibited by the NPR-A cytoplasmic domain, we fused this domain to the C terminus of the vasorin CTF13 construct, producing Vasn+NPR-A CTF13 (Figure 5H). DAPT treatment of this fusion protein resulted in a small but significant 1.8-fold increase in CTF levels (Figure 5I), demonstrating its regulation by γ-secretase. Considering that vasorin CTF13 levels increase 10-fold upon γ-secretase inhibition (Figure 4G and 4L), we hypothesize that this observed reduction in cleavage efficiency of the vasorin transmembrane domain by γ-secretase is due to partial inhibition by the NPR-A cytoplasmic domain.

### Association of Substrate and Nonsubstrate CTFs with γ-Secretase

**Figure 6:**
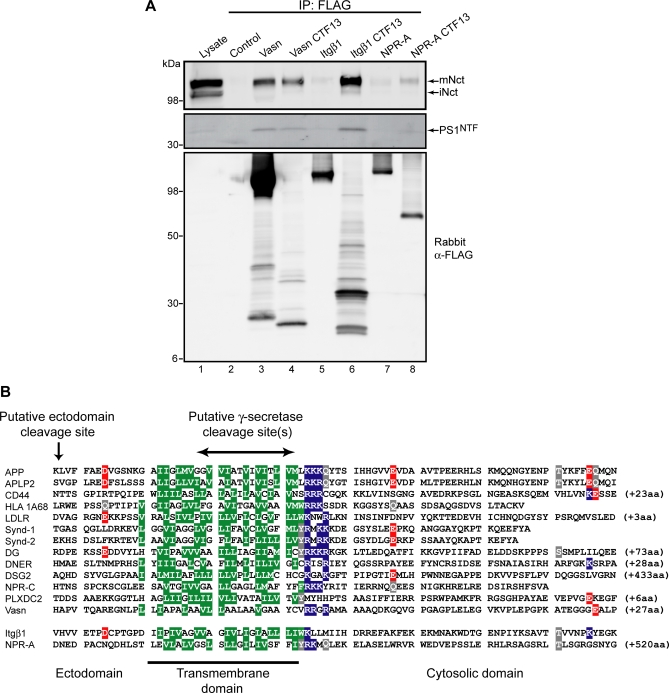
Co-Immunoprecipitation of CTFs with γ-Secretase (A) HEK cells stably expressing the indicated FLAG-tagged, full-length or CTF constructs or else untransfected control were immunoprecipitated for the FLAG epitope to probe for association with the γ-secretase complex. Immunoprecipitated samples were probed by Western blot for nicastrin (top panel), PS1 (middle panel), and the FLAG-tagged protein (bottom panel). Whole-cell lysate from control cells was run in lane 1 for comparison. (B) Amino-acid sequences of the γ-secretase substrates identified in our screen and two nonsubstrates (Itgβ1 and NPR-A; bottom), with the putative α-secretase (ectodomain) and γ-secretase cleavage sites indicated at the top. Sequences are aligned along their transmembrane domain, and amino acids showing similarity down a column are highlighted according to side chain polarity: acidic (red), basic (blue), polar neutral (grey), and nonpolar (green).

To exclude the possibility that the nonsubstrate chimeric CTFs we expressed were cleaved simply because they were in a subcellular location where no γ-secretase exists, we performed co-immunoprecipitation experiments. Previous reports indicate that substrate CTFs, and to a much lesser extent, full-length proteins prior to ectodomain shedding, associate with γ-secretase [46,47]. The initial binding by γ-secretase to a CTF may principally be regulated by nicastrin and only require that the CTF have a free N terminus [48]. We immunoprecipitated for the FLAG epitope at the C terminus of several of our stably transfected constructs (Figure 6A, bottom panel), then probed the immunoprecipitates for the co-precipitation of nicastrin (Figure 6A, top panel) and PS1 (Figure 6A, middle panel) to determine association with the γ-secretase complex. Both full-length vasorin and vasorin CTF13 were able to co-immunoprecipitate with the γ-secretase components (Figure 6A, lanes 3 and 4), as expected of these established γ-secretase substrates. By Western blot, the CTF derived from the full-length vasorin runs larger than vasorin CTF13 (Figure 6A, lower panel), suggesting that the sheddase for vasorin cleaves somewhat further than 13 amino acids from the membrane. We found that a slightly larger vasorin construct, vasorin CTF25, had a molecular weight more consistent with the natively produced CTF, and behaved similar to vasorin CTF13 (Figure S2B–S2D). Whereas full-length Itgβ1 was not able to significantly co-precipitate with nicastrin or PS, Itgβ1 CTF13 robustly associated with the γ-secretase components (lanes 5 and 6). Lysates of cells expressing Itgβ1 CTF13 have an immunoreactive band principally at the expected size, although immunoprecipitation also pulls down higher–molecular weight aggregates (Figure 6A, lower panel, lane 6). NPR-A CTF13, another nonsubstrate with a short ectodomain, was able to associate with γ-secretase to a much greater extent than full-length NPR-A (lanes 7 and 8). Co-immunoprecipitation of other substrates with γ-secretase was observed, and these results show a correlation between the amount of CTF present in the cellular lysates and the extent of association with nicastrin and PS1 (Figure S2A).

Considering that nonsubstrates are able to co-precipitate with γ-secretase, we sought to determine whether the nonsubstrate CTF chimeras can inhibit γ-secretase activity by comparing the levels of endogenous APP CTF in cells expressing substrates and nonsubstrates (Figure S3A). Under control and DAPT treatment conditions, we found no difference in APP CTFs between cells expressing substrates and nonsubstrates (Figure S3B), demonstrating that cellular expression of nonsubstrate CTFs does not interfere with endogenous γ-secretase processing. We also analyzed the processing of APP and Itgβ1 C100FLAG constructs in a purified in vitro γ-secretase activity assay. Under standard assay conditions, we detected cleavage of APP C100FLAG to generate an ICD, but we were unable to detect cleavage of Itgβ1 C100FLAG (Figure S3C). To examine the ability of Itgβ1 C100FLAG to inhibit the processing of APP, we co-incubated the two constructs together with γ-secretase and observed reduced APP ICD production (Figure S3D). Although it is unclear how this inhibition occurs, one plausible interpretation is that Itgβ1 is acting as a competitive inhibitor of APP's association with γ-secretase in this purified preparation. These data, taken together with the data in Figures 4 and 5, suggest that having a short ectodomain is sufficient to allow association with γ-secretase, but that permissive transmembrane and cytoplasmic domains are required for further processing by the protease. Furthermore, the ability of the nonsubstrate CTFs to associate with γ-secretase but not get cleaved provides further evidence for the existence of independent docking and catalytic sites within the γ-secretase complex [48,49].

Figure 6B shows partial amino acid sequences of the proteins identified in this proteomics screen, with the two nonsubstrates that we analyzed (Itgβ1 and NPR-A) listed at the bottom. Sequences are aligned along the transmembrane domains, and amino acids showing similarity down a column highlighted based on side chain polarity. In contrast to many other soluble proteases and even other intramembrane proteases [50], there is no apparent motif that predicts whether a protein is a γ-secretase substrate.

## Discussion

By performing a critical proteolytic event in several pathways of signal transduction, γ-secretase is essential for embryonic development, the maintenance of adult tissues, and the pathogenesis of certain diseases [51]. In this study, we have used an unbiased proteomic approach to identify proteins within the cell membrane that are regulated by γ-secretase. By quantitatively comparing the proteomes of cells treated with and without a potent γ-secretase inhibitor, we could identify accumulated substrates of the protease. Using tools such as small molecule inhibitors or gene silencing, the methods we applied here can, in principle, be exploited to determine the substrates regulated by any protease of interest. For enzymes similar to γ-secretase, where no substrate recognition sequence has been identified [52,53], quantitative proteomics may be the most effective unbiased method of endogenous substrate identification.

We found that among the thousands of proteins surveyed, only relatively few were processed by γ-secretase. In line with previously described substrates, all are type I transmembrane proteins whose ectodomain is shed prior to γ-secretase cleavage. Using a β-secretase inhibitor and a broad spectrum metalloprotease inhibitor, we were able to provide evidence for the proteases responsible for ectodomain shedding. For a few substrates, we found small but significant decreases in CTF levels by inhibiting α-secretase metalloproteases. That we found only a small decrease for a few substrates could have several explanations, such as the absence of a cognate ligand for the substrate to stimulate its ectodomain shedding, an incomplete inhibition of the responsible sheddase due to our obligatory use of a broad-spectrum sheddase inhibitor, or a generally low rate of ectodomain shedding of a particular substrate in the cultures under study. By choosing to focus our analysis on human HeLa cells, we limited the size and diversity of the proteome we examined, and thus it is not surprising that we did not identify all of the previously known γ-secretase substrates. Similar investigations of different cell types, or of various tissues from inhibitor-treated mice using other labeling methods [54], would greatly broaden the scale of analysis and likely reveal additional substrates. That only a select number of proteins are detected as γ-secretase substrates suggests that γ-secretase may not be an indiscriminate intramembrane protease, and that the γ processing of these substrates may have functional signaling implications. For some substrates, including Notch [55], ErbB4 [43], and N-cadherin [56], a functional γ-secretase cleavage has been clearly demonstrated, in that the respective ICDs are released from the membrane to modulate transcription. For other substrates, such as APP, the purpose of ICD production has been more elusive, but several functions have been proposed [57–59].

Several of the new substrates that we have identified could have important signaling functions regulated by γ-secretase. DNER, as a known Notch ligand and with its involvement in patterning cerebellar development, may signal antagonistically to Notch in a similar fashion as some other Notch ligands [60]. DG levels have been found to decrease in muscular dystrophy, and any deficit in signaling through the DG ICD, which is remarkably stable compared to the other ICDs we observed, may be an important and thus far unappreciated contributor to the disease's progression. Beyond promoting intercellular adhesion, desmoglein-2 has been implicated in signaling involving tumorigenesis, and expression of a recombinant cytoplasmic fragment of DSG2 has recently been reported to modulate apoptosis in intestinal epithelial cells [61]. Our finding that DSG2 can be shed by either β-secretase or an α-secretase–like metalloprotease followed by γ-secretase–mediated liberation of its ICD may identify the proteolytic pathway that generates this signaling fragment. NPR-C was previously considered an inactive clearance receptor, although some studies have found functional consequences of NPR-C signaling that are mediated by its short cytoplasmic tail [35]. Our demonstration that NPR-C is processed by γ-secretase to liberate this functional domain from the membrane may more clearly define the molecular mechanism behind NPR-C signaling, as well as raise the additional possibility of its modulating transcriptional activity by gaining access to the nucleus as an ICD. Although these proposed consequences of γ-secretase processing and ICD function for the newly identified substrates are speculative at this juncture, our description of the sequential proteolytic processing of these substrates recommends further functional studies.

The experiments with chimeric constructs provide insight into which features γ-secretase requires in recognizing and cleaving its substrates. First, the protein's ectodomain must be removed before cleavage, as previously reported [44]. For example, fusing the integrin ectodomain to vasorin's transmembrane and cytoplasmic domains precludes both sheddase and γ-secretase cleavage. However, when the large ectodomain is removed (producing the Int/Vas CTF13 constructs), these proteins are readily processed by γ-secretase. Although shortening of the ectodomain can enable binding to the γ-secretase complex (e.g., Intβ1 CTF13 and NPR-A CTF13; Figure 6A), this is not sufficient to promote proteolysis (Figures 4F and 5A). Our chimeric results suggest that the ectodomain contributes little to substrate specificity, although some reports suggest that the short ectodomain may modulate the avidity of γ-secretase processing [62,63].

Second, substrates must have both permissive transmembrane and cytoplasmic domains. Extensive mutagenesis experiments can now be performed to determine precisely what makes these domains permissive in vasorin but not in integrin, but in general, the primary sequence of proteins has thus far not been a reliable predictor of substrates of γ-secretase. Perhaps permissive domains allow the CTF to adopt a conformation amenable to γ cleavage, as is the case for substrates of the intramembrane protease rhomboid [64], but such a conformational change has yet to be examined experimentally.

Third, inhibitory domains may exist on a protein that preclude γ-secretase processing. We show this to be the case for NPR-A, whose large cytoplasmic domain reduces cleavage of the NPR-A CTF and also does so when fused to end of the NPR-C and vasorin CTFs (Figure 5). This inhibitory function is not due simply to the large size of the cytoplasmic domain; it may alternatively lie in protein folding that prevents proper entry of the transmembrane domain into the active site of γ-secretase, in a subcellular localization that protects the protein from recognition by γ-secretase, or in protein associations that anchor the cytoplasmic domain in a rigid conformation that γ-secretase does not efficiently recognize.

Finally, our data suggest that at least two pathways may exist that allow for the processing of CTFs. The first, as exemplified by vasorin, NPR-C, and other permissive CTF13 constructs, is the initial production of a CTF apart from γ-secretase that may later bind to the complex and undergo proteolysis. The second mechanism is suggested by the contrast between the full-length Vas/Int construct, which generates a CTF after ectodomain shedding in the secretory pathway and is processed by γ-secretase (Figure 4B), and Vas/Int CTF13, which shares the same size and sequence but is not processed by γ-secretase (Figure 4C). A possible explanation for this finding is that the occurrence of ectodomain shedding in situ flags the protein for γ-secretase cleavage by forcing a preferred conformation and/or by initiating a multi-protein interaction that passes the newly produced CTF directly to γ-secretase for processing. Further experimentation is now required to clarify these newly proposed mechanisms for substrate proteolysis by γ-secretase. This work should enhance our understanding of the many physiological processes regulated by this ubiquitous and conserved protease and at the same time provide insights into the proteolytic mechanism of γ-secretase that can be exploited for therapeutic advances.

## Materials and Methods

### SILAC and LC-MS/MS.

HeLa cells were propagated for six doublings in DMEM lacking l-lysine and l-arginine (Invitrogen), and supplemented with 10% dialyzed fetal bovine serum (FBS) (Calbiochem), antibiotics, and either ^12^C^14^N arginine, ^12^C^14^N lysine (“light”), or ^13^C^15^N arginine, ^13^C^15^N lysine (“heavy”) (Cambridge Isotope Laboratories). Cells were then treated with the γ-secretase inhibitor DAPT [65] (light label condition) or DMSO (heavy label condition) as control for 16 h in labeling media containing 0.5% dialyzed FBS. After treatment, cells were suspended by trituration with PBS containing 5 mM EDTA and counted. Equal numbers of cells from each treatment were combined and subjected to hypotonic lysis and Dounce homogenization. Nuclei were removed from the homogenate by spinning at 1,000*g* for 10 min, and the remaining supernatant was spun at 125,000*g* for 1 h to pellet cellular membranes. The membrane fraction was washed with 100 mM Na_2_CO_3_ (pH 11.5), briefly sonicated, and spun again at 125,000*g* for 1 h to pellet membranes.

One hundred micrograms of the purified membrane proteins were run on an 8–16% Tris-Glycine SDS-PAGE gel, stained with Coomassie blue, divided into ten molecular weight gel slices, and subject to in-gel digestion with trypsin. Liquid chromatography tandem mass spectrometry (LC-MS/MS) was performed using an LTQ FT hybrid linear (2-D) ion trap-Fourier transform ion cyclotron resonance (FTICR) mass spectrometer (ThermoFisher) as previously described [66]. Resulting MS/MS spectra were matched to a composite target-decoy [67] human sequence database [68], by both SEQUEST and Mascot search engines. An in-house algorithm was used to select confident peptide identifications with an estimated false discovery rate less than 1%. Confident peptide identifications were then subjected to Vista, an automated software suite which measures the relative abundance of light and heavy isotopic peptide pairs [14,69]. This analysis yielded over 16,400 quantitative peptide comparisons, with an estimated false discovery rate of 10%.

Proteins were considered substrates when the following conditions were met: (1) peptides derived from a higher molecular weight gel band, consisting of full-length protein, have a Light:Total peptide ratio of 0.5; (2) peptides derived from a lower–molecular weight gel band, consistent with a shorter fragment after ectodomain shedding, have a Light:Total peptide ratio of 0.65 or greater; (3) each unique peptide is identified more than once; (4) multiple unique peptides from the protein are identified.

### Cell culture and treatments.

Human embryonic kidney (HEK) 293-FT (Invitrogen) and mouse embryonic fibroblasts derived from *Presenilin1/2* null mice [21] were grown in Dulbecco's modified Eagle's medium containing 10% FBS, 2 mM l-glutamine, 100 μg/ml penicillin, and 100 μg/ml streptomycin. Transfections were performed with Fugene6 (Roche Applied Sciences). Stable cell lines were generated by transduction with lentivirus containing the cDNAs of interest, as previously described [70]. Cells were treated with the γ-secretase inhibitor DAPT (10 μM) for 6 or 16 h in Opti-MEM I (Invitrogen) to monitor CTF accumulation. HEK cells were treated for 6–16 h with the β-secretase inhibitor C3 (3 μM, BACE inhibitor IV) or the metalloprotease inhibitor GM6001 (15 μM), or for 6 h with the proteasome inhibitor epoxomicin (1 μM) or phorbol 12-myristate 13-acetate (PMA, 0.5 μM). All drugs were purchased from Calbiochem.

### Cloning.

Full-length cDNAs were obtained from the National Institutes of Health Mammalian Gene Collection. The NPR-C and Itgβ1 cDNA were from mouse, and all other cDNAs were human. Expression constructs were C-terminally tagged with the FLAG epitope (DYKDDDDK) by inserting the sequence encoding FLAG into the 3′ primer before the stop codon. To generate artificial CTFs, the construct's signal peptide and putative CTF region were individually amplified by PCR with overlapping complimentary regions added to the primer. These two overlapping amplicons were then combined and PCR amplified with outside primers, as previously described [70]. Chimeric constructs were similarly produced using primers designed with overlapping regions. All expression constructs were verified by DNA sequencing.

### Immunoblotting and immunoprecipitation.

Cells were lysed in 50 mM Tris-HCl (pH 7.4), 1% NP-40, protease inhibitor cocktail (Roche Applied Sciences), 2 mM 1,10-phenanthroline and 5 mM EDTA. Lysate was centrifuged at 1,000*g* for 10 min to remove nuclei. Protein concentrations were determined using a bicinchoninic acid-based assay (Pierce Biotechnology). Samples were then subjected to SDS-PAGE and Western blotting. APP was detected using the polyclonal antibody C9 (1:1,000) [71]; nicastrin with N1660 (1:2,500, Sigma); presenilin with MAB1563 (1:1,000, Chemicon); and FLAG tag with M2 (1:1,000, Sigma) or Rabbit anti-FLAG (F7425 1:1,000, Sigma). Western blots were probed with anti-mouse, anti-rabbit, or anti-rat secondary antibodies (1:10,000, Rockland Immunochemicals) and detected using the Odyssey infrared imaging system (LI-COR Biosciences). For co-immunoprecipitation experiments, cells were lysed in 1% CHAPSO with 25 mM Tris-HCl (pH 7.4), 100 mM NaCl, 2 mM EDTA and protease inhibitor cocktail. FLAG-tagged proteins were immunoprecipitated overnight with an M2 Affinity Gel (Sigma) and subjected to three washes with lysis buffer containing 0.5% CHAPSO before Western analysis. Immunoblots shown are representative of at least three experiments. Immunoreactive proteins were quantified using Odyssey Software v1.2 and the data were analyzed using a one-way analysis of variance with Tukey post-hoc comparison or a two-tailed Student *t*-test, where appropriate. Calculated comparisons of *p* < 0.05 were considered significant. All reported values represent the means ± standard error of the mean (SEM).

### In vitro γ-secretase assay.

Purification of γ-secretase from the S-1 CHO cell line and preparation of recombinant substrates was performed as previously described [72]. Recombinant APP and Itgβ1 C100FLAG-tagged proteins consist of an N-terminal start codon (methionine) and a C-terminal FLAG tag joined to the 99-residue CTF, and were produced by bacterial expression and subsequent purification. APP and Itgβ1 have cytoplasmic domains of identical length. Purified γ-secretase was incubated with lipids (phosphatidylcholine and phosphatidylserine) and the C100FLAG substrate at 37 °C for 4 h. The reaction was assayed by Western blotting for the C-terminal FLAG tag. For co-incubation experiments where APP and Itgβ1 C100FLAG proteins were mixed, half the normal amount of each protein was combined so as to keep the total concentration of protein and detergent consistent.

## Supporting Information

Figure S1 Quantitative Substrate CTF Changes Arising from Inhibitor Treatments (A–H) CTF levels were determined from Western blots like those shown in Figure 3 and compared to the vehicle (DMSO) condition. (B) DG CTF accumulation after 16-h treatment of HEK stably transfected cells (left graph) and HeLa stably transfected cells (right graph).
(C and D) Effects of 16-h inhibitor treatment on DG CTFs expressed in HEK cells. In both DG and DNER cells, a prolonged 16-h treatment with C3 produced a small but significant increase in CTF levels compared to DAPT control.
(I) Altered morphology of HEK cells stably expressing vasorin (scale bar = 200 μm). For comparisons to the control (DMSO) condition, *p < 0.05, **p < 0.01, ***p < 0.001.
(4.58 MB TIF)

Figure S2 Co-Immunoprecipitation of Novel Substrates with γ-Secretase (A) HEK cells stably expressing FLAG-tagged full-length substrates were immunoprecipitated and probed for association with γ-secretase as in Figure 6.
(B) Co-immunoprecipitation of vasorin and recombinant vasorin CTFs with γ-secretase. The principal band of vasorin CTF25 co-migrates with natively produced vasorin CTF.
(C and D) Accumulation of vasorin CTF25 upon γ-secretase inhibition. The fold accumulation of CTFs arising from DAPT treatment versus DMSO control; ***p < 0.001.
(E) Co-migration of natively produced NPR-C CTF with NPR-C CTF13.
(4.73 MB TIF)

Figure S3 Effects of Nonsubstrates on γ-Secretase Processing in Cells and In Vitro (A) Western blot of endogenous APP CTF levels in cells stably expressing recombinant CTF substrates or nonsubstrates in the presence or absence of DAPT.
(B) APP CTFs are significantly elevated by DAPT but are unaffected by the expression of a nonsubstrate. The fold accumulation of CTFs arising from DAPT treatment versus DMSO control; ***p < 0.001.
(C) In vitro γ-secretase activity assay measuring cleavage of APP C100FLAG and Itgβ1 C100FLAG proteins.
(D) Co-incubation of the nonsubstrate Itgβ1 C100FLAG and the APP C100FLAG proteins with γ-secretase reduces the production of the APP ICD.
(3.27 MB TIF)

Table S1 Peptides Unchanged by γ-Secretase Inhibition (3.60 MB XLS)

Table S2 Putative γ-Secretase Substrates Identified by Proteomics (49 KB XLS)

## Abbreviations

Aβ: amyloid β-protein
APP: β-amyloid precursor protein
CTF: C-terminal fragment
DG: dystroglycan
DNER: Delta/Notch-like EGF-related receptor
DSG2: desmoglein-2
HEK: human embryonic kidney
HLA: human leukocyte antigen
ICD: intracellular domain
Itgβ1: integrin beta-1
MW: molecular weight
NPR: natriuretic peptide receptor
NTF: N-terminal fragment
PLXDC2: plexin domain-containing protein 2
PS: presenilin
PS-DKO: presenilin double-knockout
PMA: phorbol 12-myristate 13-acetate
SILAC: stable isotope labeling with amino acids in cell culture
